# Clinical significance of bone marrow involvement by immunoglobulin gene rearrangement in *de novo* diffuse large B-cell lymphoma: a multicenter retrospective study

**DOI:** 10.3389/fonc.2024.1363385

**Published:** 2024-02-12

**Authors:** Yu Ri Kim, Ho Jin Shin, Ho-Young Yhim, Deok-Hwan Yang, Yong Park, Ji Hyun Lee, Won-Sik Lee, Young Rok Do, Yeung-Chul Mun, Dae Sik Kim, Jin Seok Kim

**Affiliations:** ^1^ Division of Hematology, Department of Internal Medicine, Yonsei University College of Medicine, Gangnam Severance Hospital, Seoul, Republic of Korea; ^2^ Division of Haematology-Oncology, Department of Internal Medicine, Pusan National University School of Medicine, Busan, Republic of Korea; ^3^ Division of Haematology-Oncology, Department of Internal Medicine, Jeonbuk National University Medical School, Jeonju, Republic of Korea; ^4^ Division of Haematology-Oncology, Department of Internal Medicine, Chonnam National University Hwasun Hospital, Jeollanam-do, Republic of Korea; ^5^ Division of Hematology-Oncology, Department of Internal Medicine, Korea University Anam Hospital, Seoul, Republic of Korea; ^6^ Department of Internal Medicine, Dong-A University College of Medicine, Busan, Republic of Korea; ^7^ Division of Haematology-Oncology, Department of Internal Medicine, Inje University Busan Paik Hospital, Busan, Republic of Korea; ^8^ Division of Hemato-Oncology, Department of Internal Medicine, Keimyung University Dongsan Medical Center, Daegu, Republic of Korea; ^9^ Department of Internal Medicine, Ewha Women’s University College of Medicine, Seoul, Republic of Korea; ^10^ Division of Hematology-Oncology, Department of Internal Medicine, Korea University Guro Hospital, Seoul, Republic of Korea; ^11^ Division of Hematology, Department of Internal Medicine, Yonsei University College of Medicine, Severance Hospital, Seoul, Republic of Korea

**Keywords:** diffuse large B-cell lymphoma, bone marrow involvement, immunoglobulin gene rearrangement, progression-free survival, transplantation

## Abstract

**Background:**

Bone marrow (BM) involvement is an indicator of a poor prognosis in diffuse large B-cell lymphoma (DLBCL); however, few studies have evaluated the role of immunoglobulin gene rearrangement (IgR) in detecting BM involvement.

**Methods:**

We evaluated the clinical characteristics and treatment outcomes of patients with DLBCL based on histological BM involvement or positive BM IgR using polymerase chain reaction or next-generation sequencing. We also investigated the role of consolidative upfront autologous hematopoietic stem cell transplantation (ASCT) in patients with DLBCL and BM involvement.

**Results:**

Among 624 patients, 123 (19.7%) with histological BM involvement and 88 (17.5%) with positive IgR in histologically negative BM had more advanced disease characteristics. Overall (OS) and progression-free (PFS) survival was better for patients with negative BM histology and negative IgR than that in patients with histological BM involvement (*P* = 0.050 and *P* < 0.001, respectively) and positive IgR with negative BM histology (*P* = 0.001 and *P* = 0.005, respectively). Survival rates did not differ among 82 (13.1%) patients who were treated with upfront ASCT and had histological BM involvement or positive IgR with negative BM histology. The survival outcomes were worse for patients who were not treated with upfront ASCT and for those with histological BM involvement or positive IgR, than for those with negative BM histology and negative IgR.

**Conclusion:**

Patients diagnosed with DLBCL and BM involvement based on histology or IgR had aggressive clinical features and poor survival. Upfront ASCT mitigated poor prognosis due to BM involvement.

## 1 Introduction

Although the treatment outcomes of diffuse large B-cell lymphoma (DLBCL) have improved with the development of new drugs, relapse is frequent and associated with dismal outcomes (1, 2). Bone marrow (BM) involvement is classified as extranodal and stage 4, which increases the international prognostic index (IPI) and is directly linked to shorter survival (3–5). The reported incidence of BM involvement is 11%-36% and the classic definition of BM involvement is abnormal lymphoma cells in BM aspirates or biopsies (3, 4, 6). However, minimal BM involvement of malignant lymphoma cells often generates false negative results because a histological diagnosis is very difficult in the absence of significant morphological changes (7–9). Bone marrow involvement can be diagnosed using 18F-FDG PET, but only within a limited range, and diagnostic rates vary depending on the lymphoma subtype (10–13). These problems have been addressed using the polymerase chain reaction (PCR) to detect immunoglobulin gene rearrangement (IgR) in BM samples because B-cell non-Hodgkin lymphoma (NHL) undergoes clonal IgR (9, 14). Clonal immunoglobulin heavy chain (IGH) and kappa chain (IGK) gene rearrangement could help the diagnostic process when histological findings are inconclusive. Moreover, gene rearrangement can be a helpful indicator during follow-up, as well as for diagnoses (15, 16). Patients with negative histological BM can be classified based on whether they test positive for IgR and negative for BM histology which indicates a more accurately determined advanced stage and a poorer prognosis (14, 17). Immunoglobulin gene rearrangement has mostly been detected using PCR; however, next-generation sequencing (NGS) has also been recently used (18). Detecting BM involvement in patients newly diagnosed with DLBCL indicates poor prognosis; to that end, IgR tests have been applied in a few studies to detect BM involvement in patients DLBCL treated with R-CHOP (rituximab, cyclophosphamide, doxorubicin, vincristine, and prednisolone) chemotherapy (14, 19–21).

Patients newly diagnosed with DLBCL accompanied by negative BM histology and poor outcomes of current standard treatment should be tested for IgR to precisely diagnose BM involvement. New treatment approaches should also be applied such as high-intensity chemotherapy to overcome BM involvement as a poor prognostic factor (5). Here, we investigated the clinical characteristics and treatment outcomes of upfront consolidative ASCT as part of a high-intensity chemotherapeutic regimen in patients with DLBCL and BM involvement determined by histological or molecular biological methods.

## 2 Materials and methods

This study enrolled patients from nine institutions in Korea who were newly diagnosed with DLBCL based on the World Health Organization classification (22) and histological BM involvement between 2010 and 2019. The study was conducted according to the guidelines of the Declaration of Helsinki, and approved by the Institutional Review Board of Severance Hospital (4-2019-0579 Aug 5, 2019) and each institution.

The control group comprised patients from Severance Hospital who were newly diagnosed with DLBCL and were tested for IgR regardless of BM involvement status within the same period. The exclusion criteria were disease transformation from indolent follicular lymphoma, primary central nervous system lymphoma, cutaneous DLBCL, primary mediastinal B-cell lymphoma, and human immunodeficiency virus-associated DLBCL. All patients were administered with R-CHOP as first-line chemotherapy. Upfront consolidative ASCT was considered for patients with Ann Arbor stages III or IV and elevated lactic dehydrogenase (LDH) levels who achieved complete (CR) or partial (PR) remission after R-CHOP chemotherapy. The international prognostic index (IPI) score was calculated as described (23). Responses were assessed based on the Cheson criteria (24).

### 2.1 Histological diagnosis of bone marrow involvement

We obtained aspirates and BM biopsies from the posterior superior iliac crest from all enrolled patients before starting chemotherapy for DLBCL. Bone marrow involvement was diagnosed based on histological criteria and immunochemical staining for B-cell markers (3, 8). Concordant BM involvement was defined as BM involvement of DLBCL, while discordant involvement was defined as involvement of small and low-grade lymphoma cells (3). The present study investigated only concordant BM involvement. Thirteen patients had discordant BM involvement without DLBCL involvement, and these patients were classified as negative. Cells of origin were classified based on the Hans algorithm using immunochemical staining (25).

### 2.2 Immunoglobulin gene rearrangement test to diagnose bone marrow involvement

We assessed clonal gene rearrangement in BM aspirates from 504 patients. The assays included BIOMED-2 multiplex primer sets in five master mixes that targeted the IGH and two master mixes that target the IGK locus. Fragment analysis was applied to fluorescence-labeled PCR products using an ABI 3130 DNA sequencer (Thermo Fisher Scientific Inc., Waltham, MA, USA) and GeneMapper 3.2 software (Thermo Fisher Scientific Inc.). Next-generation sequencing (NGS) was applied from April 2017 using LymphoTrack^®^ IGH FR1 and IGK Assays (Invivoscribe Technologies Inc., San Diego, CA, USA). After PCR amplification, libraries were purified using the Agencourt AMPure XP system (Beckman Coulter, Inc., Brea, CA, USA). Quantified libraries were sequenced on a MiSeq system using MiSeq Reagent Kit v2 (Illumina Inc., San Diego, CA, USA). Bioinformatics were analyzed using LymphoTrack^®^ Dx MiSeq Data Analysis version 2.4.3 (Invivoscribe Technologies, Inc.). The cut-offs for clonality and clonotype sequences were determined as described by the manufacturers. We assessed IgR in patients without histological BM involvement. Positive IgR was defined as positive IGH and/or IGK gene rearrangement. The sensitivity of PCR is 10^-3^ and that of NGS is 10^-4^.

### 2.3 Statistical analysis

Overall survival (OS) was determined as elapsed time between the dates of diagnosis and death, regardless of the cause. Surviving patients were censored at the last date of follow-up. Progression-free survival (PFS) was defined as elapsed time between the dates of diagnosis to progression, relapse, or death from any cause. Survival was analyzed using Kaplan-Meier curves, and pairs of groups were compared using log-rank tests. A Cox proportional hazard model was used for multivariate analysis. The multicollinearity of all variables in univariate analyses was assessed as tolerance and a variance inflation factor using linear regression analysis. Values with *P* < 0.05 in all analyses were considered statistically significant. All data were statistically analyzed using SPSS for Windows, version 23.0 (IBM Corp., Armonk, NY, USA).

## Results

### 2.4 Patients’ characteristics

**Figure 1:**
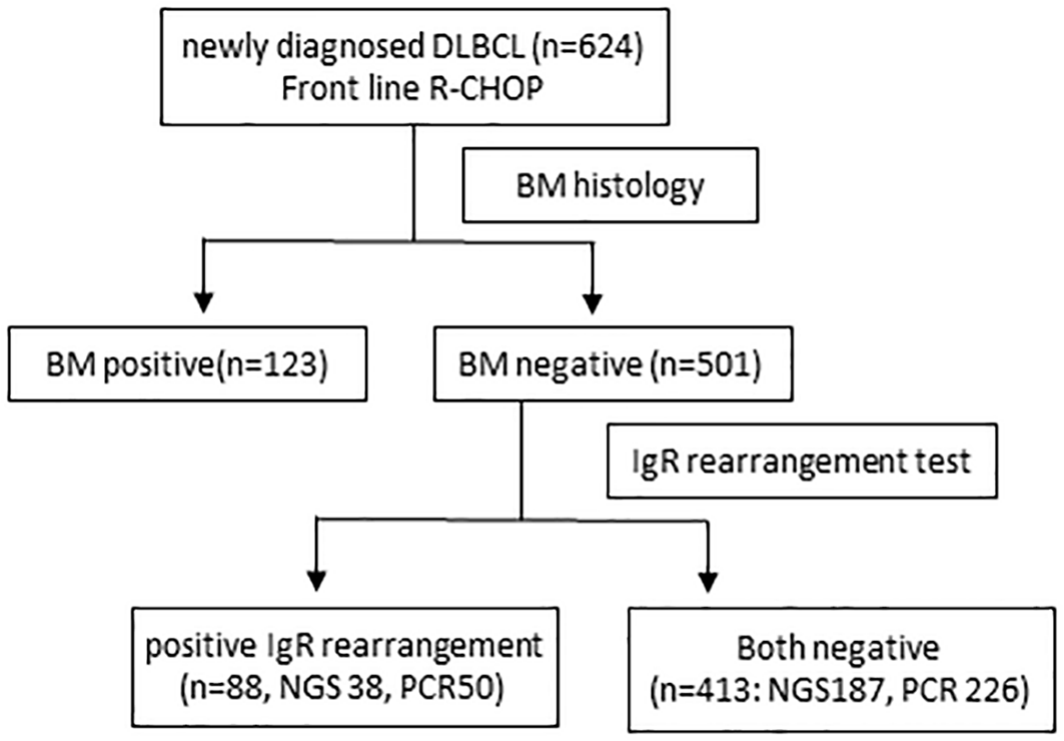
Flowchart of patients. BM, bone marrow; DLBCL, diffuse large B-cell lymphoma; IgR, immunoglobulin gene rearrangement R-CHOP, rituximab, cyclophosphamide, doxorubicin, vincristine, and prednisolone.

Among 624 patients newly diagnosed with DLBCL, 123 (19.7%) had histological BM involvement. Among 501 patients without histological BM involvement, 88 (17.5%) were IgR positive, 29 (5.7%) and 26 (5.1%) had positive IGH and IGK rearrangement, respectively, and 33 (6.5%) had rearranged IGH and IGK (**Figure 1**). Patients with histological BM involvement or positive IgR with negative BM histology tended to be older (*P* = 0.02 and *P* < 0.001, respectively). Moreover, these patients had advanced-stage DLBCL with extranodal involvement at more than one site and elevated LDH, as well as significantly higher IPI scores than patients who were negative for both (**Table 1**). We tested 276 (55.1%) patients for clonal IgR using PCR. Fifty (50/276, 18.1%) patients showed positive results by PCR, whereas 38 (16.9%) of 225 patients had positive results of NGS. The rates of positivity rates did not significantly differ between the two test methods (*P* = 0.814).

**Table 1:** Clinical characteristics of 624 patients according to BM involvement or immunoglobulin gene rearrangement.

Characteristics	BM negative (n=413) No. (%)	BM positive (n=123) No. (%)	Clonal IgR^a^ (n=88) No. (%)	Comparison between two factors p-value		
				BM negative vs BM positive	BM negative vs clonal IgR	BM positive vs clonal IgR
Age, median (range) years	61 (19–86)	56 (19–85)	66 (41–89)	0.02	<0.001	<0.001
Male patients	236 (57.1)	64 (52.0)	54 (61.4)	0.352	0.479	0.206
ECOG 2-4	28 (6.8)	28 (22.8)	15 (17.0)	<0.001	0.005	0.387
Stage III or IV	177 (42.9)	123 (100)	58 (65.9)	<0.001	<0.001	<0.001
Extranodal sites >1	114 (27.6)	93 (75.6)	38 (43.2)	<0.001	0.005	<0.001
LDH, elevated	186 (45.0)	110/122 (90.2)	54/88 (61.4)	<0.001	0.007	<0.001
Non-GCB subtype	278 (67.3)	62/93 (66.7)	66/88 (75.0)	0.903	0.166	0.254
DEL	102 (24.7)	21/74 (28.4)	36/88 (40.9)	0.561	0.004	0.102
Upfront ASCT	19 (4.6)	53 (43.1)	10 (11.4)	<0.001	0.022	<0.001
IPI				<0.001	<0.001	<0.001
Low/Low-intermediate	293 (70.9)	26 (21.1)	43 (45.3)			
High-intermediate/High	120 (29.1)	97 (78.9)	52 (54.7)			

a This patient was IgR positive and BM histology negative. ASCT, autologous hematopoietic stem cell transplantation.

BM, bone marrow; DEL, double expressor lymphoma; ECOG, Eastern Cooperative Oncology Group;IgR, Immunoglobulin gene rearrangements; GCB, germinal center B-cell; IPI, International prognostic index; LDH, Lactate dehydrogenase.

### 2.5 Treatment outcomes according to bone marrow involvement

**Figure 2:**
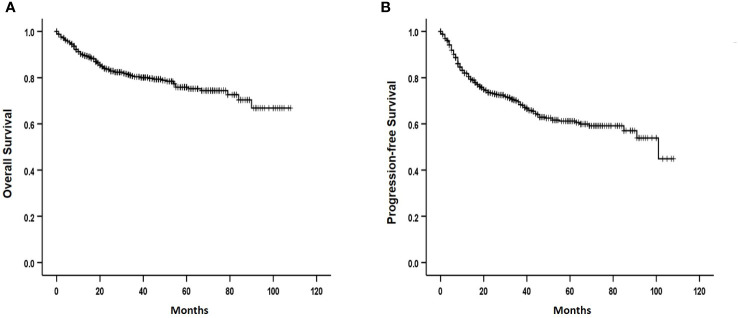
Overall **(A)** and progression-free **(B)** survival of patients.

**Figure 3:**
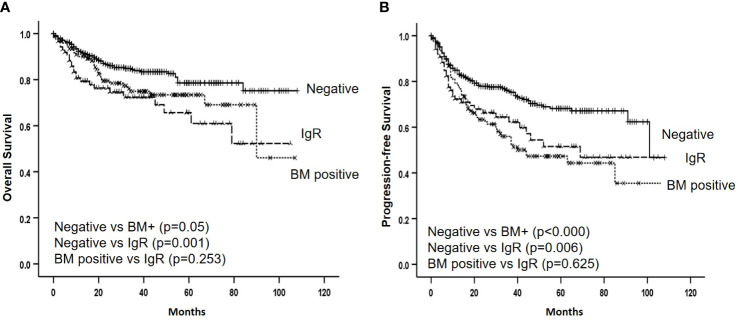
Overall **(A)** and progression-free **(B)** survival according to bone marrow involvement by histology and immunoglobulin gene rearrangement. BM, bone marrow, IgR, immunoglobulin gene rearrangement.

Among the registered patients who received R-CHOP chemotherapy as the first-line treatment, 587 responded. A CR was achieved in 465 (79.2%) of 587 evaluable patients, which included 93 (78.2%) of 119) with histological BM involvement. These findings did not significantly differ from those of patients without BM involvement (*P* = 0.428). Meanwhile, 56 (70.0%) of 80 evaluable patients with positive IgR achieved CR. This was significantly lower than the 316 (76.5%) of 388 patients without histologic and molecular BM involvement (*P* = 0.032). The median follow-up was 32 (range: 1-108) months, and the 3-year OS and PFS rates were 80.4% and 69.5%, respectively (**Figures 2A, B**). The 3-year OS and PFS were 74.9% and 56.0% in patients with histological BM involvement and 72.4% and 62.4% in those with positive IgR and negative BM histology. These were lower than the survival outcomes of patients with negative IgR and BM histology (83.9% and 75.7%, respectively; (**Figures 3A, B**).

### 2.6 Treatment outcomes according to autologous hematopoietic stem cell transplantation

**Figure 4:**
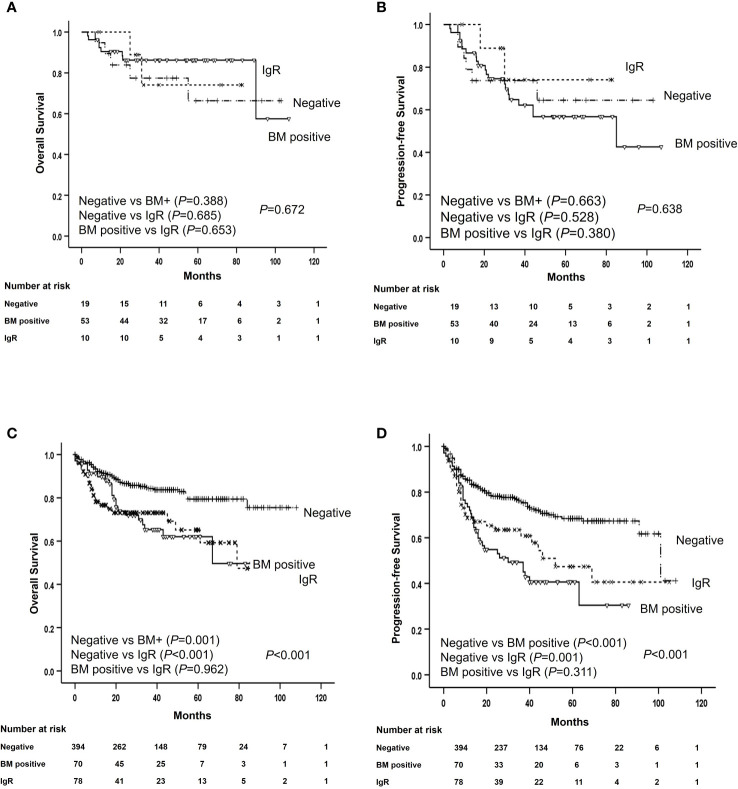
Survival of patients with and without ASCT according to histological bone marrow involvement and IGR. Overall and progression-free survival of patients with **(A, B)** and without **(C, D)** ASCT according to histological bone marrow involvement and immunoglobulin gene rearrangement. ASCT, autologous stem cell transplantation; BM, bone marrow; IgR, immunoglobulin gene rearrangement; OS, overall survival; PFS, progression-free survival.

Upfront consolidative ASCT was administered to 82 (13.1%) patients after they completed frontline R-CHOP chemotherapy. Among them, 53 (64.6%) had histological BM involvement, 10 (12.2%) had positive IgR and negative BM histology, and 19 (23.2%) did not have histological BM involvement and were IgR negative. Treatment outcomes were analyzed according to upfront ASCT in patients with advanced-stage and elevated LDH levels. The OS and PFS rates were better for patients who were administered upfront ASCT than for patients who were not (*P* = 0.010 and *P* = 0.004, respectively). The OS and PFS outcomes of patients who received upfront ASCT to minimize selection bias associated with treatment intensity did not significantly differ according to histological BM involvement (*P* = 0.388 and *P* = 0.663, respectively) or positive IgR (*P* = 0.685 and *P* = 0.528, respectively; **Figures 4A, B**). The 3-year OS and PFS rates among patients who did not receive upfront ASCT were poorer for those with BM involvement than for those without (65.0% *vs*. 85.1%, *P* = 0.001, and 49.2% *vs*. 77.0%, *P* < 0.001, respectively). The OS and PFS rates were also lower for patients with positive IgR than for those without histological BM involvement and negative IgR (72.0% *vs*. 85.1%, *P* < 0.001 and 60.6% *vs*. 77.0%, *P* < 0.001, respectively; **Figures 4C, D**).

### 2.7 Univariate analysis of prognostic factors associated with poor survival

We assessed the results of the univariate analysis of patients who were not treated with upfront ASCT. The following factors were significantly associated with poor prognosis: age ≥ 60 years, poor performance status, advanced disease stage, involvement of at least one lymph node, elevated LDH, histological BM involvement, positive IgR, non-germinal center B-cell (GCB) subtype, and IPI. The multivariate analysis associated poor OS and PFS with elevated LDH (*P* < 0.001 and *P* = 0.001, respectively), poor performance status (*P* = 0.004 and *P* = 0.05, respectively), and positive IgR (*P* = 0.001 and P = 0.004, respectively; **Table 2**). In contrast, age was the only prognostic factor among patients who received upfront ASCT (P = 0.001 and P = 0.036, respectively).

**Table 2:** Prognostic factors of OS and PFS among patients who did not receive upfront ASCT.

	OS		PFS	
Univariate analysis				
**Variable**	HR (95% CI)	p-value	HR (95% CI)	p-value
**Age ≥60**	1.890 (1.253-2.852)	0.002	1.683 (1.227-2.308)	0.001
**Male**	1.026 (0.694-1.516)	0.898	1.287 (0.941-1.760)	0.114
**ECOG ≥2**	3.474 (2.183-5.531)	<0.001	2.918 (1.973-4.315)	<0.001
**Stage 3,4**	2.207 (1.463-3.331)	<0.001	3.015 (2.150-4.226)	<0.001
**Extranodal >1**	2.501 (1.697-3.686)	<0.001	3.067 (2.257-4.167)	<0.001
**LDH, elevated**	3.706 (2.344-5.860)	<0.001	2.856 (2.046-3.987)	<0.001
**BM involvement**	1.929 (1.203-3.094)	0.006	2.211 (1.539-3.179)	<0.001
**IgR**	2.699 (1.752-4.159)	<0.000	2.289 (1.608-3.259)	<0.001
**GCB vs non-GCB**	1.501 (0.966-2.333)	0.071	1.533 (1.082-2.174)	0.016
**DEL**	1.149 (0.734-1.799)	0.544	1.066 (0.749-1.517)	0.722
**IPI**	3.282 (2.202-4.894)	<0.000	3.404 (2.491-4.651)	<0.001
Multivariate analysis				
**IgR**	2.145 (1.379-3.337)	0.001	1.711 (1.186-2.468)	0.004
**LDH, elevated**	2.862 (1.760-4.655)	<0.001	1.887 (1.292-2.755)	0.001
**ECOG≥2**	2.200 (1.379-3.737)	0.004	1.589 (1.000-2.524)	0.050
**Extranodal >1**			2.128 (1.480-3.061)	<0.001

BM, bone marrow; CI, confidence interval; ECOG, Eastern Cooperative Oncology Group; GCB, Germinal center B-cell; HR, hazard ratio; IgR, Immunoglobulin gene rearrangement; DEL, double expressor lymphoma; IPI, International prognostic index; LDH, lactic dehydrogenase.

## 3 Discussion

The present study findings revealed that the clinical characteristics of patients with DLBCL and positive IgR in BM samples (besides those with traditional histological BM involvement) who received R-CHOP chemotherapy, resembled those of patients with advanced-stage lymphoma. Moreover, these patients did not respond well to R-CHOP first-line treatment and had poor OS and PFS. Therefore, tests for IgR should be applied to precisely predict the prognosis of patients with negative BM histology.

BM involvement of DLBCL cells showed an unfavorable gene signature, which was related to tumor cell proliferation, migration, and immune escape. These could explain high-risk clinical features and poor prognosis (5). However, differentiating the histological diagnosis of BM involvement of malignant lymphoma cells can be challenging particularly in patients with small amount of lymphoma cells. The IgR test could be helpful under such circumstances. The IgR results were positive in 13%–16% of patients with DLBCL who were diagnosed with histologically normal BM and these patients did not survive for long (14, 20). Here, we found positive IgR in 17.5% of patients with negative histological BM involvement, which was similar to previous findings. Without IgR tests, these patients would have been classified as having no BM involvement and the disease stage would have been lowered. Therefore, routine IgR tests of BM samples should be recommended to evaluate the molecular BM involvement of DLBCL cells.

A higher proportion of patients with histological BM involvement had a more advanced disease stage, more frequent extranodal involvement, and more elevated LDH than patients with positive IgR and negative BM histology. Patients with histological BM involvement were classified as having stage 4 disease or a high IPI score at the time of diagnosis. However, patients with positive IgR might not be classified as having an advanced disease stage and might have been down-staged because histological BM involvement was not found. Therefore, the clinical characteristics of patients with histological BM involvement differed from those with only positive IgR with negative BM histology. Nevertheless, we found that the differences in OS and PFS between patients with histological BM involvement and those with positive IgR and negative BM histology were not significant. In addition, the multivariate analysis identified positive IgR as an important prognostic factor associated with poor OS and PFS in patients who were not treated with upfront ASCT.

The most useful tool for assessing clonality in patients with NHL until recently was BIOMED-2 PCR assays. These had been widely used as they were standardized and deemed suitable for technically routine test environments (26). However, PCR is limited by being unsuitable for samples with poor DNA quality, such as formalin-fixed paraffin-embedded (FFPE) samples, which could produce false negative results (15, 18). However, small amplicons and FFPE samples can be analyzed using NGS (18). We compared the ability of PCR and NGS to detect clonality and found no significant differences.

Although histological BM involvement is considered a poor prognostic factor, a standardized treatment approach has not yet been established. Furthermore, patients with positive IgR have not been studied. High-intensity chemotherapy, such as fractionated cyclophosphamide, vincristine, doxorubicin, and dexamethasone alternating with high-dose methotrexate and cytarabine (rituximab-hyper-CVAD/MA) or dose-adjusted etoposide, prednisone, vincristine, cyclophosphamide, doxorubicin, and rituximab (EPOCH-R) might overcome poor prognoses, and treatment outcomes are better than those of R-CHOP in high-risk patients with DLCBL (5). Consolidative upfront ASCT might also be considered as a different approach to high-intensity chemotherapy for DLBCL because it can eradicate PCR-detectable NHL cells and consequently reduce recurrence (27). Upfront ASCT in the rituximab era improves PFS in high-risk patients with DLBCL (28, 29). Based on this, we investigated whether upfront ASCT could mitigate the poor prognosis of patients with DLBCL and BM involvement. According to Korean reimbursement guidelines, consolidative upfront ASCT in clinical practice can be recommended for patients with elevated LDH and stage III/IV DLBCL at the time of diagnosis who respond to front-line R-CHOP chemotherapy. However, the present study was retrospective, and as a result, patients with good treatment response and performance might have been selected to receive upfront ASCT. Accordingly, the patients were divided into groups with and without upfront ASCT when analyzing the prognostic factors associated with survival to minimize bias associated with the intensity of treatment. Analysis of all enrolled patients showed that survival was shorter for patients with histological BM involvement or positive IgR with negative BM histology than for those without histological BM involvement and negative IgR. The results of the multivariate analysis showed that patients with poor performance status, elevated LDH, or positive IgR who did not receive upfront ASCT tended to have poor OS and PFS. These results indicated that upfront ASCT plays an important role in overcoming a poor prognosis due to histological BM involvement or positive IgR with negative BM histology. The routine application of upfront ASCT consolidation after R-CHOP is not considered standard care in all countries. However, we suggest that upfront ASCT for high-risk patients with DLBCL and BM involvement should be considered at least in those countries with access to novel target agents.

This study had the following limitations. First, this was a retrospective study and not a prospective randomized study. To overcome this limitation, we registered as many patients as possible from nine institutions in Korea. Another limitation was the absence of regular follow-up data for IgR tests, although they were applied at the time of diagnosis. Based on the concept of minimal residual disease, follow-up tests for IgR are underway and will be examined through further follow-up studies. Although IgR tests could not discriminate infiltration by a high- or low-grade component, the poor prognostic impact of IgR positivity for patients with DLBCL nevertheless generated meaningful information. Despite these limitations, our findings were meaningful insofar as we used PCR and NGS to investigate the role of IgR, in addition to histological BM involvement, in a large cohort of patients with DLBCL and analyzed their clinical characteristics and treatment outcomes.

In conclusion, tests to detect IgR BM allowed a more detailed classification of the prognosis of patients who were negative for histological BM involvement. Patients who did not receive upfront ASCT could not overcome the poor prognosis associated with BM involvement. Our results suggested that ASCT could mitigate the poor prognosis of not only patients with histological BM involvement but also those with positive IgR and negative BM histology. Accordingly, these findings require validation through future prospective studies.

## Data availability statement

The raw data supporting the conclusions of this article will be made available by the authors, without undue reservation.

## Ethics statement

The studies involving humans were approved by Ethical Review Committee of Severance Hospital. The studies were conducted in accordance with the local legislation and institutional requirements. The ethics committee/institutional review board waived the requirement of written informed consent for participation from the participants or the participants’ legal guardians/next of kin because this is a retrospective cohort study.

## Author contributions

YK: Conceptualization, Data curation, Formal analysis, Funding acquisition, Investigation, Methodology, Resources, Software, Validation, Visualization, Writing – original draft, Writing – review & editing. HS: Data curation, Resources, Writing – original draft, Writing – review & editing. H-YY: Data curation, Resources, Writing – original draft, Writing – review & editing. D-HY: Data curation, Resources, Writing – original draft, Writing – review & editing. YP: Data curation, Resources, Writing – original draft, Writing – review & editing. JL: Data curation, Resources, Writing – original draft, Writing – review & editing. W-SL: Data curation, Resources, Writing – original draft, Writing – review & editing. YD: Data curation, Resources, Writing – original draft, Writing – review & editing. Y-CM: Data curation, Resources, Writing – original draft, Writing – review & editing. DK: Data curation, Resources, Writing – original draft, Writing – review & editing. JK: Conceptualization, Data curation, Formal analysis, Funding acquisition, Investigation, Methodology, Project administration, Resources, Software, Supervision, Validation, Visualization, Writing – original draft, Writing – review & editing.
